# Primary extraskeletal myxoid chondrosarcoma in cerebellum: A case report with literature review

**DOI:** 10.1097/MD.0000000000008684

**Published:** 2017-11-27

**Authors:** You Qin, Hai-bo Zhang, Chang-Shu Ke, Jing Huang, Bian Wu, Chao Wan, Chen-Su Yang, Kun-Yu Yang

**Affiliations:** a Cancer Center, Union Hospital, Tongji Medical College, Huazhong University of Science and Technology, Wuhan, Hubei; b Zhejiang Provincial People's Hospital, Hangzhou, Zhejiang; c People's Hospital of Hangzhou medical college, Hangzhou, Zhejiang Province; d Department of Pathology, Tongji Hospital, Tongji Medical College, Huazhong University of Science and Technology, Wuhan, Hubei, China.

**Keywords:** brain neoplasm, cerebellum neoplasms, extraskeletal myxoid chondrosarcoma, temozolomide

## Abstract

**Rationale:**

Extraskeletal myxoid chondrosarcoma (EMC) is a rare malignant neoplasm of which intracranial EMC is the rarest.

**Patient concerns:**

We present an unusual case report of a 41-year-old woman who was sent to the emergency department for a sudden headache and other symptoms related to increased intracranial pressure.

**Interventions:**

Emergent CT revealed an occupying lesion in the left cerebellum with surrounding edema. A complete surgical excision of the lesion through a transcortical approach was performed. After the operation, this patient received adjuvant radiotherapy and temozolomide treatment.

**Diagnoses:**

Pathology diagnosis was an intracranial EMC.

**Outcomes:**

The patient survives with no tumor recurrence as of the last follow-up. Progression-free survival exceeded 20 months.

**Lessons:**

We have reviewed the literature and here summarize the diagnosis and treatment options for intracranial EMC. Diagnosis and treatment options of this rare disease are discussed.

## 1 Introduction

Extraskeletal myxoid chondrosarcoma (EMC) is a very rare malignant neoplasm. Only 12 cases of intracranial EMC have been reported since the condition was first described in 1972 (Table 1). Of these, only 2 cases were located in the cerebellum.^[1]^

**Table 1:**
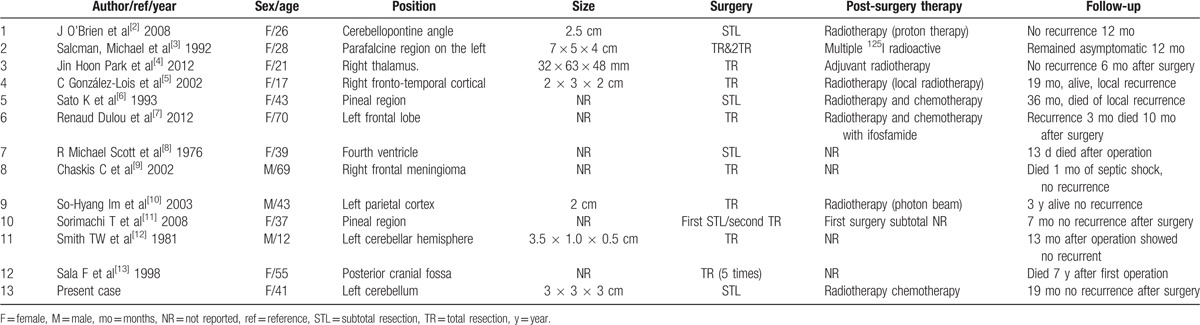
Reported cases of primary intracranial extraskeletal myxoid chondrosarcoma.

EMC was first recognized by Stout and Verner in 1953. Nineteen years later, Enzinger and Shiraki^[1]^ defined it as a distinct clinicopathological entity in 1972. It usually occurs in the deep soft tissues of the extremities. According to a multi-institutional study of 42 cases in Japan, 29 cases of EMC (69%) were located in the lower extremities.^[14]^ It is quite rare for a patient to show detectable neurological symptoms upon initial presentation.

Here, we have presented a case of intracranial EMC located in the cerebellum. Because of the exiguity of intracranial EMC, we believe this case report may be important to surgeons and oncologists. Collecting and organizing such rare case reports is the first step toward characterizing this tumor and improving our knowledge of this rare disease. We have reviewed literature and have summarized diagnosis and treatment options.

## 2 Case report

In March 2015, a 41-year-old woman presented with a sudden headache associated with vomiting of gastric contents in the afternoon and was immediately sent to the hospital. She reported no coma, convulsions of the limbs, or inability to move. Her heart rate was 73 beats/min, blood pressure was 104/65 mm Hg, and her respiratory rate was 23 breaths/min. She was breathing ambient air with satisfactory air oxygen saturation. There were no lesions in the oropharynx, and her neck was supple. The lungs were clear, and her heart rate was regular, without any murmur. Physical and neurological examination at that time revealed no special abnormalities. Her medical history revealed a sulfanilamide allergy but no other diseases.

**Figure 1:**
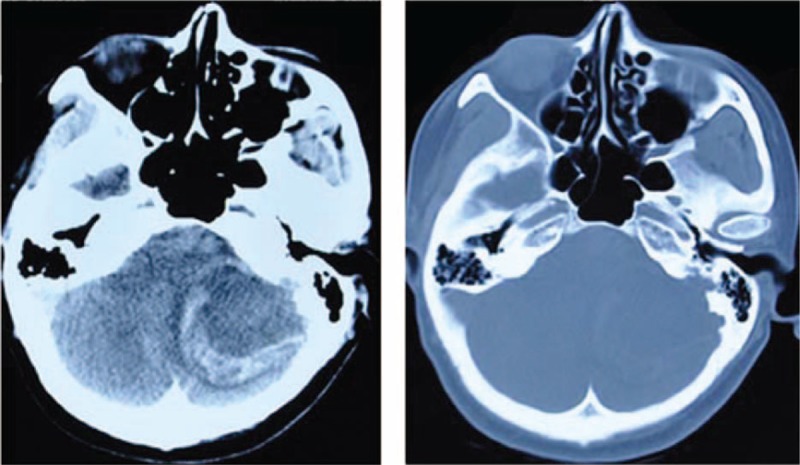
Emergent CT images showed a mass (about 4.9 × 4.3 cm) in the left cerebellum with surrounding bleeding and edema. There was a destruction of occipital bone close to tumor.

The emergent CT scan of her brain showed an occupying lesion about 4.9 × 4.3 cm in the left cerebellum accompanied by intracranial vascular bleeding. There was a destruction of occipital bone. The mass was noncalcified and slightly dense with severe peritumoral bleeding and edema (Fig. 1). We did not perform MRI because of limited time and objective conditions.

The next day, this patient suddenly fell into a coma. We then performed an operation to excise the lesion, clear the intracranial hematoma, and performed ventricular drainage and tracheotomy. We used a transcortical approach to the left cerebellum. In the operation, we performed a left parietal craniotomy and made a Y-shaped incision in the dura under a microscope. We removed a 3 × 3 × 3 cm cystic lesion with poor dissection margins from the surrounding tissue and sent it for pathologic examination. The tumor was cystic and full of clear, yellow lipid matter, with attachments to the meninges and the occipital bone. The immediate postoperative course was uneventful.

Unfortunately, a postoperative intracranial infection developed, and the patient presented with shivering and hyperpyrexia. The cerebrospinal fluid (CSF) tested positive for a Gram-positive bacterial infection and targeted antibiotic was used, which caused the patient's condition to gradually improve.

**Figure 2:**
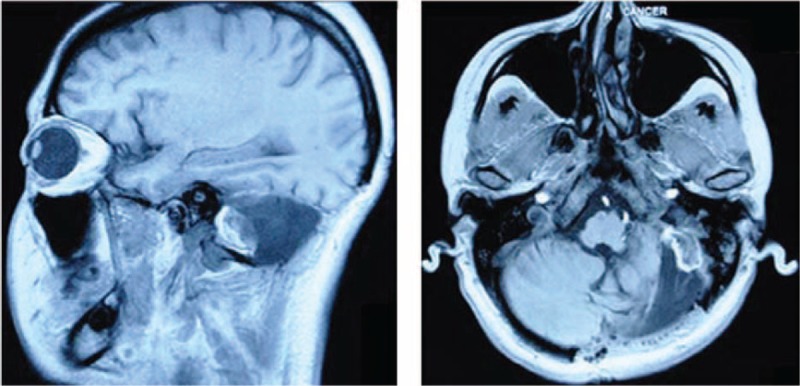
Magnetic resonance images showed the changes after surgery. Axial and coronal T1-weighted MR images revealed cerebellar cortical defect and destruction of left occipital bone. T1-weighted MR images showed long T1 low FLAIR signal cyst about 1.7 × 1.2 cm, considering as a post-operation hemorrhage.

MRI and CT scans were performed for follow-up. After surgery, MRI revealed that parts of the cerebellum and occipital lobe were absent, and a 1.7 × 1.2 cm long T1 low FLAIR cyst was attributed to a subdural hematoma (Fig. 2). Luckily, there was no abnormal mass outside the brain.

After recovering from the operation, the patient was transferred to the oncology department for antitumor treatment. Because of the bone destruction, the patient accepted radiotherapy with DT Pctv1 56Gy/28F Pctv2 50Gy/25F in the tumor bed area concurrent with chemotherapy with temozolomide. Temozolomide was taken at a dose of 150 mg/m^2^/d for 5 days in the first cycle and 200 mg/m^2^/d for 5 days in the remaining 5 cycles. The patient tolerated combination therapy well and did not experience severe side effects. After therapy, the tumor did not recur. The patient was given a CT/MRI and hematological index every 3 months and experienced no adverse events. Progression-free survival (PFS) lasted more than 20 months.

**Figure 3:**
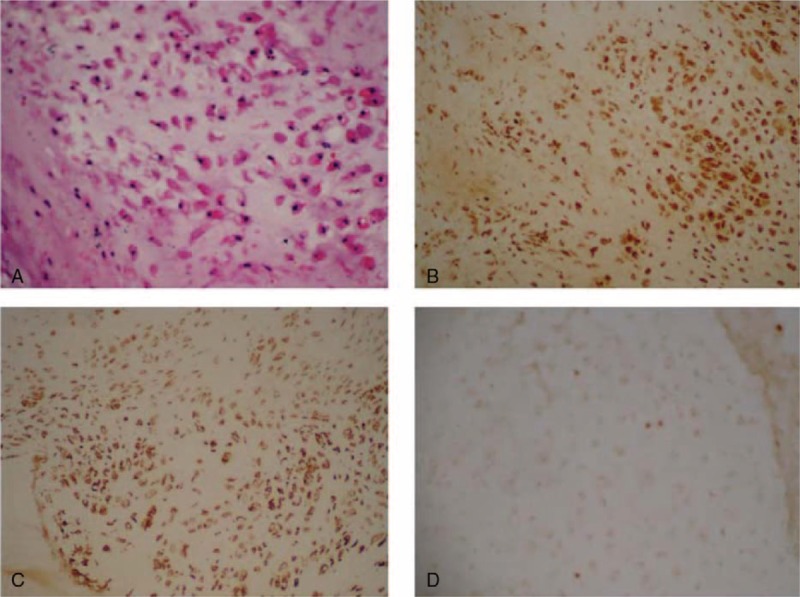
The microphotographs showed the histopathological feature of the tumor. (A) The oval or plump spindle tumor cells are loosely distributed within the myxoid chondroid matrix. The tumor cells contain the eosinophilic cytoplasm, with mild cellular atypia. H & E, original magnification ×200. (B) The tumor cells are positively stained by immunohistochemistry (IHC) of S100. Original magnification ×100. (C) The tumor cells are positively stained by IHC of Vimentin. Original magnification ×100. (D) The IHC of Ki-67 shows the labeling index as <1%. Original magnification ×100.

Pathologic diagnosis for this patient was complicated and difficult. The department of pathology of Wuhan Union Hospital diagnosed it as EMC, and immunohistochemistry (IHC) showed IHC:S100(+), PCK(−), EMA(−), cdponin(−), p63(−), GFAP(−), Nestin(−), and Ki-67(+2%) (Fig. 3). This conclusion was supported by a consultation from the Department of Pathology of Tongji Hospital. The Beijing Neurosurgical Institute also diagnosed it as EMC of mucoid cells using the cartilage matrix and immunohistochemical results. The ethics committee of Union Hospital, Tongji Medical College approved the case. All participants provided informed consent.

## 3 Review of literature and discussion

We report an EMC located in the left cerebellum, which was treated with X-beam irradiation and temozolomide after resection.

Cartilaginous tumors make up only 0.16% of all cranial/intracranial-occupying lesions.^[15,16]^ These arise from the base of the skull and have an extradural location.^[2]^ Histologically, chondroid tumors can be divided into chondromas, chondrosarcomas, myxoid chondrosarcomas, and mesenchymal chondrosarcomas. Chondrosarcoma is a rare cartilaginous neoplasm, accounting for <0.5% of all intracranial neoplasms.^[17]^

Although the name implies that its origin is from the cartilage, there is no clear evidence for the chondroid lineage of the malignant cells.^[18]^ There is still confusion about the exact site of origin in cases of intracranial EMC cases, and researchers have offered different opinions. Scott reported that the tumors arose from the stroma of the choroid plexus.^[8]^ Hassounah M found that about 50% of the cranial/intracranial chondrosarcomas typically arise from the base of the skull, where the chondrocranium formed from the fusion of a number of separate cartilages.^[19]^ Drilon supposed that intracranial chondrosarcomas originate from embryonic remnants of the chondrocranium or from metaplasia of meningeal fibroblasts and grow along the base of the skull.^[20]^ Chaskis C reported a similar hypothesis that EMCs probably originate from primitive multipotential mesenchymal cells because some cases have no attachment to the cranium or the meninges, and lesions located above the base of the skull are rare.^[9]^

It is difficult to draw a definitive conclusion from the literature about the exact origin of tumors in intracranial EMC. According to their location, it is possible that the tumors in reported cases are derived from meningeal fibroblasts in which the tumor is attached to the falx, tentorial, or dural sinuses. In patients with intraparenchymal locations, it is hypothesized that the site of origin could be the leptomeningeal sheath around vessels or the vessel walls in the depth of a sulcus.^[7]^ In this case, we assumed that the origin of the tumor was multipotential mesenchymal cells, perhaps embryonic rests, because most of the volume was in the depth of the cerebellum, with a spherical shape, and the center of the area of origin was presumed to be in the deep portion of the cerebellum.

EMC is characterized by the differentially expressed gene *CHI3L1* and the chromosome translocation involving t (9;22)(q22; q12) and translocation-generated disorder involving *EWS-TEC*, *TAF2N-TEC*, and *TCF12-TEC*.^[21]^ This special characteristic may play an important role in the development, progression, and metastasis of the neoplasm. Therefore, there is the potential for early diagnosis and treatment.

At the time of this patient's diagnosis, there was no significant sex predilection for chondrosarcoma according to a review of 50 cases (23 male and 27 female). However, EMC has significant sex predilection characteristics, with a male-to-female ratio of 2:1 and a median age at presentation of 52 (6–89). Tumor sizes range from 1 to 25 cm, with a median of 7 cm.^[7,22]^ However, reported intracranial EMC cases had a different male-to-female ratio of approximately 1:3 and a median age at presentation of 39 (12–70); the tumor sizes ranged from 0.5 to 7 cm. More cases must be examined before the significance of the male-to-female ratio can be determined; the sex ratio may offer some clues regarding the role of sex in the origination and development of intracranial EMC.

EMC has a specific histological appearance, and it is well known that the varied histological features make it difficult for precise diagnosis, even within a single tumor. EMC is a very rare malignant neoplasm, with malignant chondroblast-like cells seen in a background of myxoid matrix.^[18]^ EMC histological characteristics always include rich mucus production and a cordlike or lacelike arrangement of small round cells or short spindle cells. They also have eosinophilic cytoplasm distributed in a gelatinous background. There were some atypical EMCs, which showed a high-cellularity component, with spindle cells or homogeneous round cells. Ultrastructural studies have revealed additional differences between myxochondrosarcoma and mesenchymal chondrosarcoma, which showed that intracytoplasmic glycogen of myxochondrosarcoma cells is abundant, while mesenchymal chondrosarcoma is devoid of intracytoplasmic glycogen. The stroma of mesenchymal chondrosarcomas is rich in collagen fibrils. Myxochondrosarcoma does not share this characteristic.^[19]^

Analyses of previous immunohistochemical studies of intracranial myxoid chondrosarcomas have shown that EMCs expressed vimentin (89%), epithelial membrane antigen (28%), and synaptophysin S-100 (17%).^[2,4,23]^ The immunoreactivity of the S-100 protein and vimentin were strongly positive in the majority of EMC tumor cells, and no immunoreactivity for cytokeratin was seen.^[6]^

Regarding the difficulty of pathological diagnosis, Hiroko Noguchi developed a new diagnostic method and proposed using EWSR1 and NR4A3 probe fluorescence in situ hybridization to diagnose EMC, regardless of the presence of typical or atypical histological characteristics.^[24]^ Further studies into the relationship between morphologic features and genetic abnormalities are needed.

Because of its rarity, it is difficult to define an optimal therapeutic strategy for EMC.^[10]^ There is no doubt that the first choice for intracranial EMC is total resection. The extent of surgical resection and the ideal plan before operation is very important for EMC patients.^[3]^ The relationship between surgery and recurrence has yet to be established concretely. In an emergency, the patient receives an unplanned excision. Satoshi Kawaguchi analyzed 42 EMC patients, finding that inadequate initial surgery was significantly closely associated with poorer recurrence-free survival than definitive initial surgery.^[14]^ However, Renaud Dulou presented an intracranial EMC case that showed that total removal of the tumor with functionally safe wide excision is not sufficient to prevent short-term recurrence, and its prognosis remains particularly poor.^[7]^ In our opinion, there is a relationship between the range of the operation and tumor progression, and it is quite difficult to reach a definitive conclusion about intracranial EMC patients. More cases are needed.

Fortunately, the total operation is not difficult if well planned because of the characteristics of the margins of this kind of tumor. Intracranial EMC adhesions are not severe and always have clear margins from the surrounding tissue.^[3,5]^ Sometimes, EMC was not clearly demarcated at its deep margins during dissection from the cerebral cortex.^[7]^

Surgery is the most important type of treatment for EMC. Postoperative therapy is also quite important to prevent recurrence and progression of the intracranial EMC. Adjuvant therapies, such as radiotherapy, brachytherapy, and proton beam treatment, have been found to improve patients’ follow-up results.^[4]^ According to these reported cases, we can speculate that the radiotherapy of intracranial EMC was considered to play an important role in the management of tumor progression and prognosis.^[19,11]^ For this reason, in EMC, radiotherapy is considered especially suitable for those who are not able to receive total excision or planned excision. There is no definite radiotherapy plan with respect to dose, fraction, or type of radiation. Salcman reported that the effectiveness of conventional radiation therapy is unclear, and high doses or high-energy treatment may offer an advantage over conventional fractionated radiation.^[3]^ It is not clear whether stereotactic body radiation is suitable for these intracranial EMC patients. More clinical data or experiments to find the optimal radiation strategy are still needed.

There has been no report concerning the advantages of adjuvant chemotherapy for intracranial EMC patients. In 1993, Kazufumi Sato first reported an intracranial EMC patient treated with chemotherapy, though it is not clear exactly which therapeutic regimen was used, and Renaud Dulou used chemotherapy with ifosfamide (4 cycles of this regimen: 1000 mg/m^2^ daily for 5 days every 3 weeks); neither showed clear clinical benefits.^[7]^ In our cases, we made an attempt to use temozolomide chemotherapy to cure this disease.

Currently, very few useful drugs are available for the treatment of patients with advanced soft tissue sarcoma. To address the lack of data reported on the place of chemotherapy in the management of intracranial EMC patients, we screened therapeutic agents used for soft-tissue sarcomas, including doxorubicin, dacarbazine, beta-interferon, and ifosfamide. However, these drugs lack significant evidence for their efficacy in intracranial locations because of the intrinsic drug resistance of the tumor and the additional theoretical obstacle of the blood-brain barrier.

We chose temozolomide because of its small size and lipophilic properties. Temozolomide is 100% bioavailable when taken orally and can cross the blood-brain barrier.^[25]^ Xavier Garcia del Muro reported a phase II trial of temozolomide as a 6-week, continuous, oral schedule in patients with advanced soft tissue sarcoma. These results showed that temozolomide was tolerated well and had activity in patients pretreated soft tissue sarcomas.^[26]^ Other clinical trials for temozolomide used for advanced soft tissue sarcoma showed prolonging of the median overall survival and time to progression (Table 2).

**Table 2:**
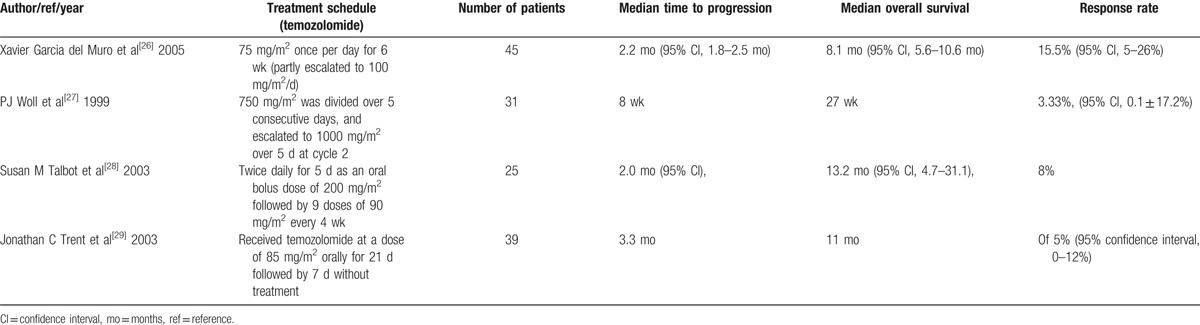
Clinical trial for temozolomide in patients with advanced soft tissue sarcoma.

For these reasons, we used the combination therapy of radiotherapy (50Gy/25F) and temozolomide 150 to 200 mg/m^2^/d as a postoperative therapy strategy. The patient's chemotherapy was withdrawn, and the patient received the best supportive care we could provide. We need new molecules and targeted agents developed for the treatment of intracranial EMC, especially those targeting molecular alterations, which could significantly improve the outcomes of patients.

Because very few cases have been reported, we knew little about the prognosis of this condition. Enzinger and Shiraki initially believed EMC to be a low-grade sarcoma with a protracted clinical course.^[1]^ However, Satoshi Kawaguchi represented EMC as an intermediate malignancy with a protracted clinical course and a high potential for metastasis.^[14]^ Renaud Dulou also regarded EMC as an intermediate-grade malignant neoplasm with a high propensity for local recurrence and metastatic evolution.^[7]^ Ernesto Bustinza-Linares pointed out that certain characteristics can cause an EMC to behave as a high-grade tumor.^[16]^ Some authors have found that EMC is highly malignant. EMC recurrence is common, and it is a much more aggressive variant of intracranial chondrosarcoma.^[3–5]^ In our opinion, intracranial EMC is an intermediate malignancy suitable for a protracted clinical course for patients who do not show any symptoms before the mass pressure affects important areas. However, there is considerable potential for local recurrence and metastasis.

Here, we attempted to identify the factors that affect EMC prognosis. Some clinical doctors have supposed that the extent of resection is the only prognostic factor.^[9]^ Some hold different opinions. The characteristics of poor prognosis include tumor sizes over 10 cm, high cellular density, the presence of anaplasia or rhabdoid features, and high mitotic activity. High Ki67 has been reported to be an adverse pathological prognostic factor.^[7,23,30,31]^ There were also other characteristics, such as there being >2/10 hpf or overall Ki67 >10%, which correlate with decreased metastasis-free survival.^[23]^

Because of the exiguity of intracranial EMC, it is difficult to collect conclusive evidence with respect to the origin, morphologic features, genetic abnormalities, therapy strategy, and prognosis. We reported an intracranial EMC patient and reviewed other cases in literature. Here, a new postoperative chemotherapy strategy was tried. Radiotherapy combined with chemotherapy or immunotherapy may a play an increasingly important role in the treatment of intracranial EMC patients.

## Acknowledgments

The authors wish to extend a sincere thanks to Prof Fu-Rong Lu and Prof Dian Wang for their assistance in modification of the language.
