# Primary extraskeletal myxoid chondrosarcoma in cerebellum: A case report with literature review: Erratum

**DOI:** 10.1097/MD.0000000000009335

**Published:** 2017-12-15

In the article, “Primary extraskeletal myxoid chondrosarcoma in cerebellum: A case report with literature review”^[1]^, which appeared in Volume 96, Issue 47 of *Medicine*, the authors would like to note that affiliation a should appear as Cancer Center, Union Hospital, Tongji Medical College, Huazhong University of Science and Technology, Wuhan 430022, China.

They would also like to note that Dr. Hai-bo Zhang should appear first in the author list.
